# Genome-wide mapping of binding sites of the transposase-derived SETMAR protein in the human genome

**DOI:** 10.1016/j.csbj.2021.07.010

**Published:** 2021-07-14

**Authors:** Márton Miskei, Adrienn Horváth, Lívia Viola, Laura Varga, Éva Nagy, Orsolya Feró, Zsolt Karányi, Jason Roszik, Csaba Miskey, Zoltán Ivics, Lóránt Székvölgyi

**Affiliations:** a MTA-DE Momentum Genome Architecture and Recombination Research Group, Department of Biochemistry and Molecular Biology, Faculty of Medicine, University of Debrecen, Hungary; b Faculty of Pharmacy, University of Debrecen, Hungary; c Department of Internal Medicine, Faculty of Medicine, University of Debrecen, Hungary; d Department of Melanoma Medical Oncology, Division of Cancer Medicine, MD Anderson Cancer Centre, University of Texas, USA; e Division of Medical Biotechnology, Paul Ehrlich Institute, Langen D-63225, Germany

**Keywords:** SETMAR/Metnase, Transposase, Histone methyltransferase, ChIP-seq

## Abstract

Throughout evolution, DNA transposons provide a recurrent supply of genetic information to give rise to novel gene functions by fusion of their transposase domain to various domains of host-encoded proteins. One of these “domesticated”, transposase-derived factors is SETMAR/Metnase which is a naturally occurring fusion protein that consists of a histone-lysine methyltransferase domain and an *HsMar1* transposase. To elucidate the biological role of SETMAR, it is crucial to identify genomic targets to which SETMAR specifically binds and link these sites to the regulation of gene expression. Herein, we mapped the genomic landscape of SETMAR binding in a near-haploid human leukemia cell line (HAP1) in order to identify on-target and off-target binding sites at high resolution and to elucidate their role in terms of gene expression. Our analysis revealed a perfect correlation between SETMAR and inverted terminal repeats (ITRs) of *HsMar1* transposon remnants, which are considered as natural target sites for SETMAR binding. However, we did not detect any untargeted events at non-ITR sequences, calling into question previously proposed off-target binding sites. We identified sequence fidelity of the ITR motif as a key factor for determining the binding affinity of SETMAR for chromosomes, as higher conservation of ITR sequences resulted in increased affinity for chromatin and stronger repression of SETMAR-bound gene loci. These associations highlight how SETMAR’s chromatin binding fine-tune gene regulatory networks in human tumour cells.

## Highlights

• SETMAR/Metnase preferentially targets *Hsmar1* transposon ends (ITRs) in living cells.
• Sequence fidelity of the ITR motif determines the affinity of SETMAR/Metnase to chromosomes.
• Higher ITR fidelity results in increased affinity for chromatin and stronger repression of SETMAR-bound gene loci.

## Graphical abstract

**Figure:**
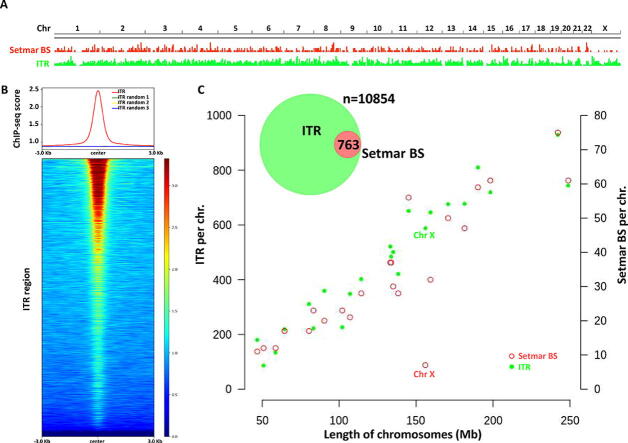

## 1 Introduction

Transposons of the *mariner* family are present in a wide variety of eukaryotic genomes, including humans [1], [2], [3]. These transposons contain a single gene encoding the transposase, flanked by short, <30-bp inverted terminal repeat (ITR) sequences. *Mariner* elements mobilize through a cut-and-paste mechanism catalyzed by the transposase, which belongs to a large family of recombinase proteins including retroviral/retrotransposon integrases and transposases, characterized by the DDE/D signature in the catalytic domain of the proteins [2], [3]. Transposition results in the accumulation of hundreds or thousands of transposon copies over evolutionary time. However, most *mariner* copies appear to be dead remnants of once active transposons inactivated by mutations [4].

**Fig. 1:**
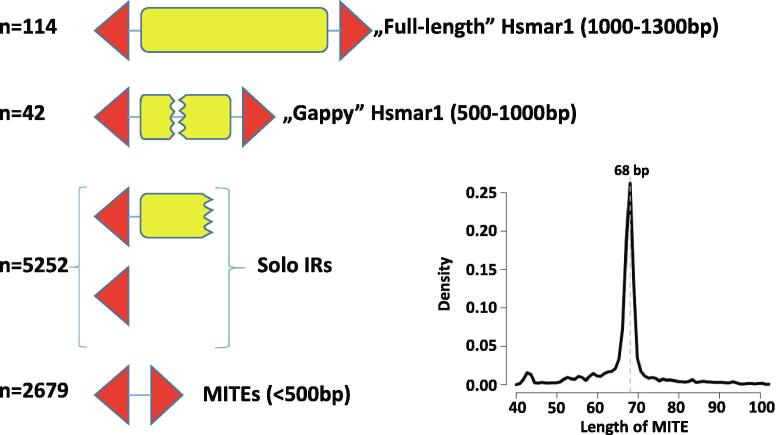
**Characteristics of *Hsmar1* mariner transposons.** “Full-length” *Hsmar1*, “gappy” *Hsmar1*, and MITEs (Miniature Inverted Repeat Transposable Elements) contain two inverted terminal repeat (ITR) sequences (red triangles). “Solo” inverted repeats contain one ITR linked to a truncated *Hsmar1* sequence or not. In each category, up to three mismatches were allowed in the flanking ITR sequences (“core” motif: 5’-GGTGCAAAAGTAATTGCGG-3’). Histogram shows the length distribution of MITE sequences with a median size of 68 bp. Only ITRs within 500 bp were considered to identify MITEs. The number of cases (n) is shown on the left. (For interpretation of the references to colour in this figure legend, the reader is referred to the web version of this article.)

*Mariner* elements are represented by two subfamilies in the human genome: *Hsmar1* [5] and *Hsmar2* [6]. The first *Hsmar1* element entered the primate genome lineage approximately 50 million years (Myr) ago, and transposition was ongoing until at least 37 Myr ago, producing 114 “full-length” *Hsmar1* copies [5] (Fig. 1. However, none of the present copies encodes a functional transposase protein due to mutational inactivation. The *Hsmar1* transposon copies are accompanied by 42 “gappy” *Hsmar1* elements containing internal deletions in their transposase coding sequences, 5252 copies of solo-ITRs (containing a single ITR) and 2679 copies of an *Hsmar1*-related, paired-ITR element, *MADE1* [5], [7] (Fig. 1). Such miniature inverted-repeat transposable elements (MITEs) are thought to have been generated by internal deletions of longer transposons (median *MADE1* length: 68 bp, Fig. 1); they make up the predominant fraction of DNA elements in flowering plants, and are often found in animal genomes [8].

Despite their parasitic nature, there is increasing evidence that transposable elements are a powerful force in gene evolution. Indeed, about 50 human genes are derived from transposable elements [7], among them genes that are responsible for immunoglobulin gene recombination in all vertebrates [9]. One of these “domesticated”, transposase-derived genes is SETMAR (also called Metnase), a fusion gene containing an N-terminal SET domain fused in-frame to an *Hsmar1* transposase [5], [10]. The SETMAR gene has apparently been under selection; the transposase open reading frame is conserved, and shows only 2.4% divergence from a consensus *Hsmar1* transposase gene sequence (*vs*. 8% average divergence between *Hsmar1* transposase genes) [5]. The SET domain can be found in histone methyltransferases that regulate gene expression by chromatin modifications [11]. Accordingly, the SETMAR protein has been shown to methylate histone H3 lysines 4 and 36 *in vitro*, and has been proposed to play a role in DNA double-strand break (DSB) repair [12].

The cellular function(s) of SETMAR remain enigmatic. Cordaux *et al*. have found that selection has been preserving the ITR-binding activity of SETMAR [10]. Accordingly, both the transposase domain of SETMAR as well as the full-length SETMAR protein were shown to bind to *Hsmar1* ITR sequences *in vitro* [10]. Thus, a function of the SETMAR protein is likely associated with its ability to specifically recognize numerous genomic binding sites represented by the *Hsmar1* ITRs. Through its ability to bind to *Hsmar1* transposon ITR sequences, and to catalyze specific histone modifications [12], SETMAR could contribute to transcriptional gene regulation by inducing targeted chromatin modifications. Indeed, *mariner* transposase domains were recently described to have a propensity to undergo domestication by recurrent fusion to host transcriptional regulatory domains, especially the Krüppel-associated box (KRAB) domain; these KRAB-transposase fusion proteins repress gene expression in a sequence-specific fashion [13].

SETMAR is broadly expressed in human tissues (Supplementary Fig. S1) and cell lines (Supplementary Fig. S2), suggesting a housekeeping function [12]. In addition, transcriptional variants of SETMAR show a broad expression pattern in human diseases including cancer [14], [15], [16]. Overexpression of SETMAR is favourable in kidney cancer and unfavourable in liver cancer, while most TCGA cancers have no significant survival association with SETMAR (https://www.proteinatlas.org/ENSG00000170364-SETMAR/pathology). Molecular explanation for these heterogeneous relationships is still unknown. To elucidate the pro- and anti-tumorigenic activities of SETMAR in a mechanistic detail, it is crucial to identify genomic targets to which SETMAR specifically binds in cancer cells and link these sites to the regulation of gene expression. A recent study used the ChIP-exo approach to map Flag-tagged SETMAR binding sites in the hyper-aneuploid U2O2 osteosarcoma cell line [17], which allowed the first evaluation of SETMAR cistrome in human tumour cells. However, the majority of ChIP-exo peaks (69% − 605 out of 875) could not be enriched at the expected target ITRs of the *Hsmar1* transposons, which are considered as natural landing sites for SETMAR chromosome binding. Significant off-target binding have been reported in another (unpublished) study [18], but the reason for SETMAR’s non-ITR binding remained unexplained. We therefore decided to map the genomic landscape of SETMAR in a near-haploid human leukemia cell line (HAP1) to identify on-target and off-target binding sites at high resolution and to elucidate their role in terms of gene expression. Our analysis revealed a perfect correlation between SETMAR and ITR sequences without any untargeted events, calling into question the previously proposed off-target regions. In addition, we identified ITR sequence conservation as a key factor for determining the affinity of SETMAR for chromosomes.

## 2 Materials and methods

### 2.1 Cell line and plasmids

The HAP1 cell line were maintained in complete Iscove’s Modified Dulbecco’s Medium (IMDM, Sigma) supplemented with 10% heat inactivated Tetracycline free Foetal Bovine Serum (iBiotech), 1% penicillin/ streptomycin (Sigma) at 37 °C with 5% CO_2_. The SETMAR knockout cell line was generated by the CRISPR/Cas9 technology. The CCTGATCATGTAGTTGGACC gRNA sequence was designed to target the endonuclease cleavage to the beginning of the 2nd exon of the SETMAR gene (chr3 4,312,904 (hg38), transcript: NM_006515). The mutated cells harbour a 10 bp deletion at the target site, which resulted in a frame shift and a premature stop codon 30 bp downstream to the cleavage locus. The generation of the knockout cell line was performed by Horizon Genomics (https://horizondiscovery.com/). The SETMAR knockout cell line was made transgenic with the *Sleeping Beauty* (SB) technology to express an N-terminally hemagglutinin (HA) tagged version of the SETMAR protein as follows. The SB transposon donor was created by blunt-end cloning the BamHI/XbaI fragment of pcDNA-HA/SETMAR to the SalI/NotI site of the pTOV-T11-SV40puro [19]. 500 ng of the resulting SB transposon donor plasmid, pTOV-HA-SETMAR-puro, was co-transfected with 100 ng of pcGlobinSB100X transposase expressing vector [20] with polyethylene imine into the knockout HAP1 cells, which were subjected to 1 μg/ml puromycin selection to obtain the polyclonal HA-SETMAR-expressing cell line. The expression of the HA-SETMAR transgene and doxycycline inducibility were verified with Western-blot analysis using anti-HA antibody (11867423001, Roche).

### 2.2 SETMAR induction

pTOV-HA-SETMAR-puro was induced by increasing doxycycline concentrations (0.2 µg/ml, 0.5 µg/ml, 1 µg/ml, 2 µg/ml) 24 h before the ChIP measurement. The HA-tagged SETMAR protein is hereinafter referred to as “SETMAR-HA” throughout the text. Expression levels were quantified by western blot and a concentration of 0.5 µM doxycycline was selected for subsequent NGS (ChIP-seq) experiments. For western blot, whole cell extracts were prepared by RIPA Buffer (50 mM Tris pH8, 150 mM NaCl, 1%, NP-40, 0.5% sodium deoxycholate, 0.1% SDS) supplemented with protease inhibitors (Pierce Protease Inhibitor Mini Tablets, Thermofisher A32953) followed by sonication (Diagenode Bioruptor® Plus; 1x5 cycles 30 sec on/off High mode). The extracts were prepared from dox-treated (induced) and untreated (uninduced) cells at a concentration of 10^7^ cells/ml. After centrifugation at 15.000 × g (at 4 °C for 20 min), protein concentrations were measured by the BCA assay (Pierce BCA Protein Assay Reagent; Thermofisher 23225). 30 µg of protein extracts were run by SDS-PAGE (5–12%AA gel, Bio-Rad MiniProtean) and transferred to nitrocellulose membranes (Millipore, Billerica, MA). Ponceau S staining was used to quantify the protein levels. Membranes were blocked with 1% BSA/PBST for 1 h and then incubated with a rabbit polyclonal anti-HA tag antibody (Abcam ab9110; 1:4000) and an anti-beta-actin mouse monoclonal antibody (8H10D10, Invitrogen; 1:2000) at 4 °C overnight, with gentle shaking. A custom-made anti-SETMAR polyclonal antibody (ThermoFisher Scientific) was also used to detect endogenous- and HA-tagged SETMAR, which was produced by immunizing rabbits with the purified protein corresponding to the C-terminal 135 amino acids of SETMAR. After three washes in 1% BSA/PBST, secondary antibodies were added at room temperature for 1 h: Alexa Fluor 647 goat anti-rabbit IgG (Invitrogen 1:1000, A-21245), and Alexa Fluor® 680 Goat Anti-Mouse IgG (1:1000, ab175775, Abcam). Fluorescent signals were detected by the Molecular Imager®PharosFX™ system (Bio-Rad).

### 2.3 Chromatin immunoprecipitation and qPCR

2x10^7^ cells were fixed by 1% formaldehyde (V = 20 ml) for 10 min at room temperature (in T175 flasks), and were then quenched by 416 mM glycine for 5 min. After three washes in ice cold PBS/T, cells were scraped off and pelleted by centrifugation at 1000xg for 5 min at 4 °C. Cell pellets were stored at −80 °C. For ChIP, 2x10^7^ cells were suspended in 1.5 ml of ChIP Lysis Buffer (50 mM HEPES-KOH pH 7.5, 140 mM NaCl, 1 mM EDTA pH8, 1% Triton X-100, 0.1% Sodium Deoxycholate, 1% SDS, supplemented with protease inhibitors) and disrupted by Fast prep (speed: 6 m/s; time: 40 sec; 2 cycles; pause time: 120 sec). Chromatin lysates were sonicated in 1.5 ml LowBind tubes (600 μl sample/tube using Diagenode Bioruptor Plus (2x5 cycles, 30 sec ON/OFF LOW). After sonication, cell debris were pelleted by centrifugation at 16.000 g (at 4 °C for 20 min). Fragment length distribution of sonicated samples (50 μl) was checked by 1% agarose gel electrophoresis after reverse crosslinking (at 65 °C for 6 h) and phenol–chloroform extraction. Immunoselection was performed by 100 µl Dynabeads™ Protein G precoated with 8 µg of rabbit polyclonal anti-HA antibody (Abcam ab9110, ChIP-grade). 5% of sonicated chromatin was saved as “input” while the rest (“IP”) was diluted 1:10 with IP Dilution Buffer (1% Triton X-100, 2 mM EDTA pH8, 20 mM Tris-HCL pH 8, 150 mM NaCl, supplemented with protease inhibitors) in 15 ml tubes and incubated with antibody-coated magnetic beads (at 4 °C overnight, with rotation). Beads were washed twice in low salt wash buffer (0.1 % SDS; 1% Triton X 100; 2 mM EDTA; 20 mM Tris-HCl; 150 mM NaCl), high salt wash buffer (0.1% SDS; 1 % Triton X 100; 2 mM EDTA; 20 mM Tris-HCl; 500 mM NaCl), LiCl wash buffer (0.25 M LiCl; 1% NP40; 1% Na-deoxicolate; 1 mM EDTA; 10 mM Tris-HCl) and in TE buffer (10 mM Tris-HCl, 1 mM EDTA) at 4 °C for 1 min, using MagnaRack. IP and input samples were then eluted in 100ul elution buffer (0.1 M NaHCO_3_, 1% SDS) for 15 min at 30 °C with frequent vortexing. Supernatants were transferred to LoBind Eppendorf tubes and stored at −80 °C. Validation of SETMAR-HA binding sites (predicted by ChIP-seq analysis, see below) were performed by real-time quantitative PCR (qPCR) using a QuantStudio12KFlex machine (Applied Biosystems™) and LightCycler® 480 SYBR Green I Master (Roche) PCR reagent, following the manufacturer recommendations. Measurements were done in triplicates from two independent biological replicate experiments, and enrichment ratios were plotted as “percent of input”, corrected for dilution. qPCR primers are listed in Supplementary Table S1.

### 2.4 Illumina sequencing and bioinformatic analysis

NGS libraries were prepared by the Nugen Ovation Ultralow System V2 library preparation kit (NuGEN Technologies) following the manufacturer’s instructions. 241 million reads were sequenced (paired-end) from two independent biological replicate experiments using an Illumina Nextseq 500 machine and the NextSeq® 500/550 Mid Output Kit v2 (Illumina). 97.31% of reads were mapped on the GRCh38 (hg38) human reference genome by Bowtie2 version 2.3.4.1 [21] using default parameters. Picard was used to remove PCR duplicates from BAM files created by Samtools version 1.10 [22], applying default parameters. Repetitive segments of the genome were blacklist filtered (according to 05.05.2020, Stanford University, Anshul Kundaje Lab) and BAM files containing 185 million mapped reads were RPKM normalised using deeptools version 3.3.1 [23] applying bamCoverage processing (bin size = 100 bp; smooth length = 300 bp). MACS2 version 2.2.6 [24] was used to identify ChIP peaks from bedGraph files, applying default parameters. IP and corresponding Input data were processed in parallel. Peaks identified in Input were filtered out from IP samples using Bedtools version 2.29.0 [25]. Eleven ChIP peaks fell into unmappable segments of the hg38 reference genome and were therefore excluded from further analysis. Computer randomized peak sets were generated by Bedtools as a null model for significance tests. Blacklisted regions were excluded from random peak set generation. Annotation of SETMAR-HA chromosomal binding sites was performed according to the genomic categories of HOMER [26]. Peaks (observed and random) were extended by +/- 500 bp and their overlap ratios were determined with the appropriate annotation categories. In the pie charts, only peak summits were considered (peak sizes were not extended).

*De novo* SETMAR-HA binding motifs were identified by the MEME Suite version 5.3.0 [27] using the MEME and MAST tools. The motifs are listed in Supplementary Table S1 (MEME MAST worksheet). 763 SETMAR peaks contain the ITR consensus sequence GGTGCAAAAGTAATTGCGG identified by Cordeux R et al. [10] as an *in vitro* binding site for SETMAR. In parallel, we mapped the ITR consensus sequence by Cordeux R et al. [10] on the hg38 reference genome using Biostring and BSgenome.Hsapiens.UCSC.hg38 (R project) allowing 0–3 mismatches, and annotated the sequences based on the number of mismatches (0-3MM ITR groups; Supplementary Table S1 “ITR_10854” sheet and MEME MAST sheet). The overlap of annotated ITRs and SETMAR-HA ChIP peaks (extended by +/- 500 bp) are shown in Fig. 4, Fig. 5. Statistical analysis was performed and plots were generated by R version 3.6.3 (2020–02-29). Heatmaps and pileup plots were created by deepTools. NGS tracks were visualized by Integrative Genome Browser (IGV) version 2.8.4 [28] and JBrowse [29]. Published genomic datasets used in our analysis: RNA-seq (SRR5266566 (GSM2493886), SRR5266578 (GSM2493898) [30], ChIP-exo (GSE108773, [17]). SRA files were converted to fastq files by fastq-dump (version 2.10.4) using default parameters. RNA-seq reads were aligned to the hg38 genome by TopHat (version v2.1.1) [31] and FPKM values (Fragments Per Kilobase of transcript per Million mapped reads) were calculated by Cufflinks (version v2.2.1) [32].

## 3 Results and discussion

**Fig. 2:**
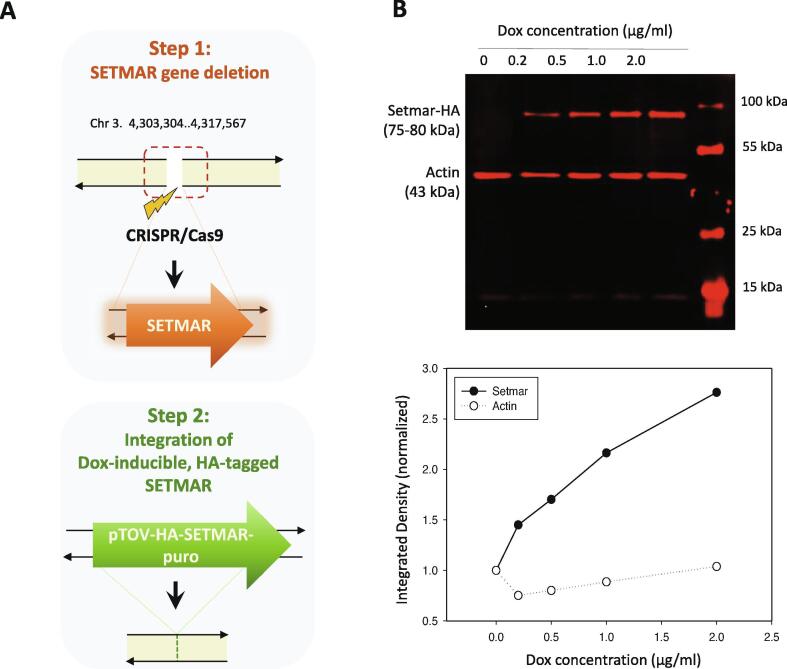
**Experimental design.** (A) Scheme of SETMAR gene deletion and integration of the conditional allele (pTOV-HA-SETMAR-puro). (B) Kinetics of SETMAR-HA induction at various tetracycline (TET) concentrations. Tet-treatment was performed for 24 h. Upper panel: western blot with anti-HA and anti-beta Actin antibodies. Lower panel: Quantification of band intensities as a function of TET concentrations.

**Fig. 3:**
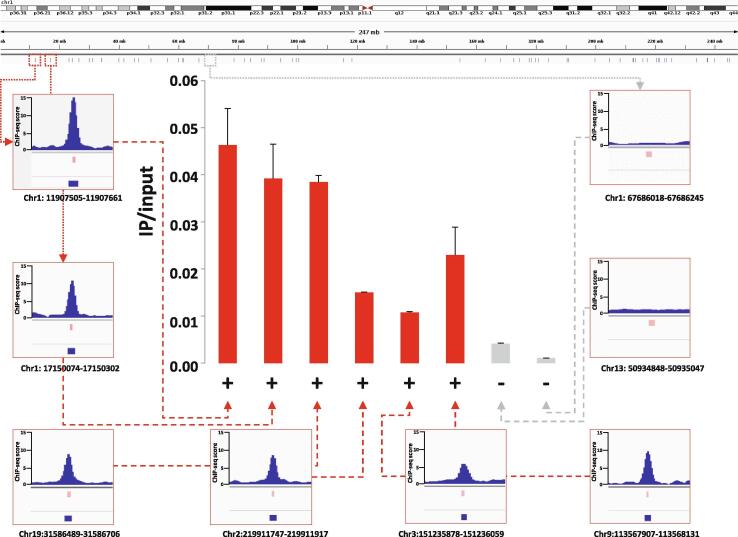
**Genome-wide mapping of SETMAR binding sites by ChIP-seq.** Upper panel: Genome browser track showing the chromosomal distribution of ChIP peaks (vertical blue bars). Lower panel: Validation of representative SETMAR-HA binding sites from different chromosomes by ChIP-qPCR. Positive and negative sites are highlighted in red and grey, respectively. Position of PCR amplicons and ChIP peak summits are shown below the IGV tracks. (For interpretation of the references to colour in this figure legend, the reader is referred to the web version of this article.)

**Fig. 4:**
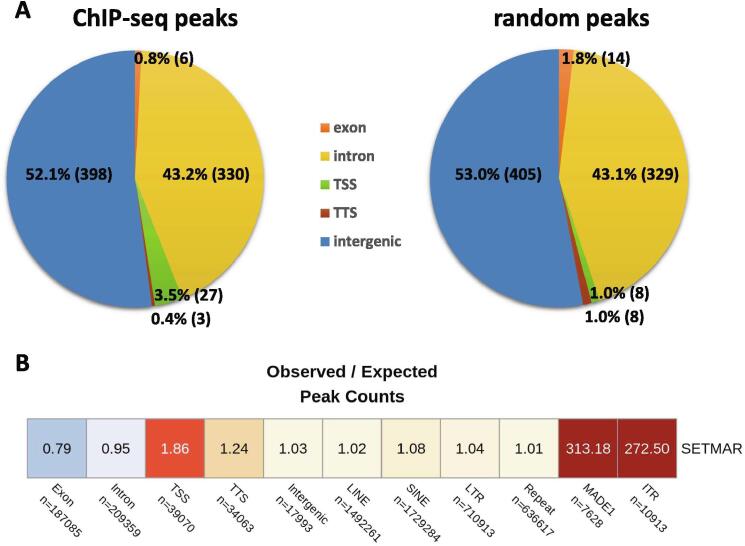
**Annotation of SETMAR-HA binding sites in the human genome.** (A) Association of ChIP peaks with genic (exon, intron, TSS, TTS) and intergenic regions. TSS: transcription start site. TTS: transcription termination site. Left and right panels: observed and expected (computer randomized) distributions, respectively. Number of peaks: 764. (B) Detailed annotation of ChIP peaks over 11 functional genomic categories. n: number of elements in categories. Cells contain observed / expected ratios for peak counts. Warmer colours represent higher enrichment. Statistically significant enrichment is observed at TSS (p-value = 0.03), *MADE1* (p-value < 2.2 × 10^-16^) and ITR regions (p-value < 2.2 × 10^-16^; prop. z test; level of significance: 0.05).

**Fig. 5:**
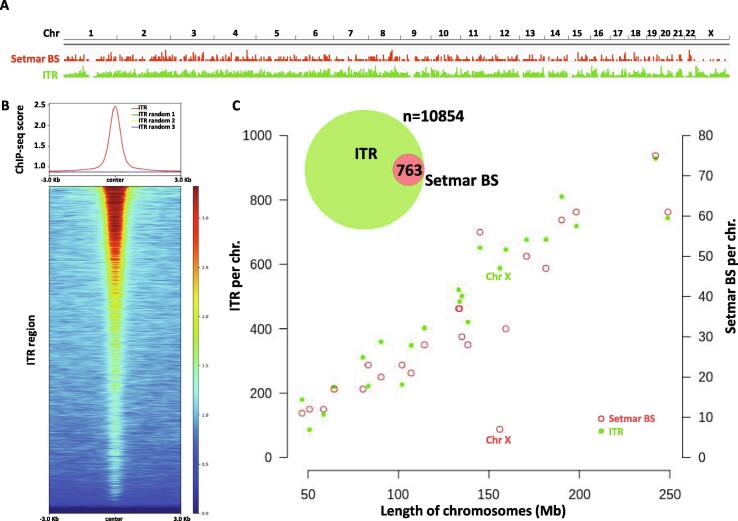
**SETMAR-HA preferentially binds to inverted terminal repeat (ITR) sequences.** (A) Genome browser track showing the distribution of SETMAR-HA ChIP peaks (blue) and ITR motifs (green) for each autosome and the sex chromosome (chrX). (B) The summit of SETMAR-HA signal perfectly coincides with ITRs (red curve and heatmap). The ChIP signal is depleted over random sites (yellow, green, and blue curves). (C) Upper panel: Proportional Venn diagram showing the overlap of SETMAR ChIP peaks and ITR motifs. Of the 764 SETMAR-HA peaks, 763 sites (99.9%) are associated with ITRs. Of the 10,854 ITRs, 1227 motifs (11.3%) are localized in SETMAR-HA peaks. Lower panel: Chromosomal distribution of SETMAR-HA binding sites and ITR motifs is strongly correlated. The number of SETMAR-HA peaks and ITRs were plotted as a function of chromosome length. They show significant covariation and correlation with chromosome length (Spearman r = 0.89; p < 0.001). Dots: chromosomes. Chromosome X represents an outlier in terms of ChIP peak numbers. (For interpretation of the references to colour in this figure legend, the reader is referred to the web version of this article.)

**Fig. 6:**
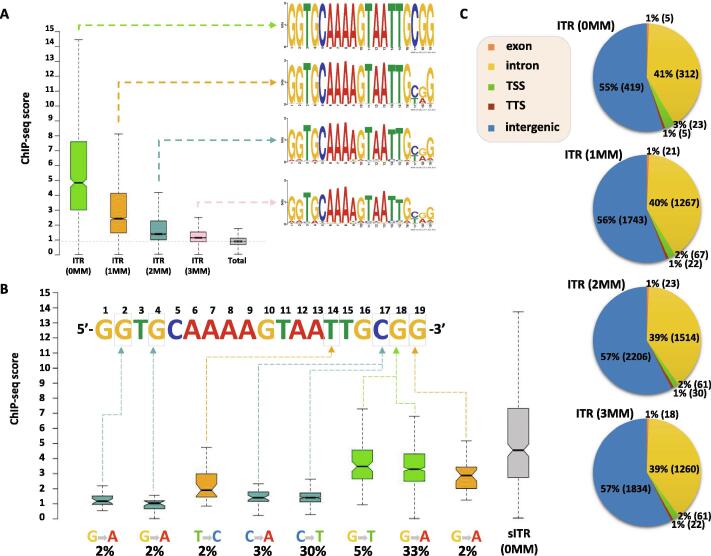
**Sequence fidelity of the ITR motif determines the affinity of SETMAR-HA binding.** (A) ITRs were grouped based on the number of mismatches in the core 19 nt motif (0MM = 763, 1MM = 3104, 2MM = 3812, 3MM = 3175), and distribution of the ChIP signal was plotted over the categories (average ChIP-seq score per ITR). “Total” (grey box) represents genomic background. There is a statistically significant difference between groups and compared to genomic background (p value < 2.2 × 10^-16^, Wilcoxon rank sum test). ChIP-seq scores, related to the affinity of SETMAR-HA binding, are inversely proportional to the number of mismatches. PWM logos are highlighted for each ITR group. (B) Identification of critical ITR nucleotide positions for SETMAR’s chromatin binding. Single-nucleotide changes that significantly reduce the affinity of SETMAR-HA for solo ITRs (sITR) are highlighted (position, mutation type, and frequency/% of cases in the 1MM group). ChIP-seq scores show a significant reduction in each group compared to 0MM sITR (p value < 10^-6^, Wilcoxon rank sum test). Number of cases: sITRs with one mismatch (1MM sITR = 1519), sITRs with zero mismatch (0MM sITR = 322). Rare events with a mutation frequency of<2% were excluded from the analysis. (C) Annotation of ITR groups in intergenic and genic (exon, intron, TSS, TTS) chromosomal regions. Percentage represents the proportion of 0-3MM ITRs within each annotation category. TSS: transcription start site. TTS: transcription termination site.

**Fig. 7:**
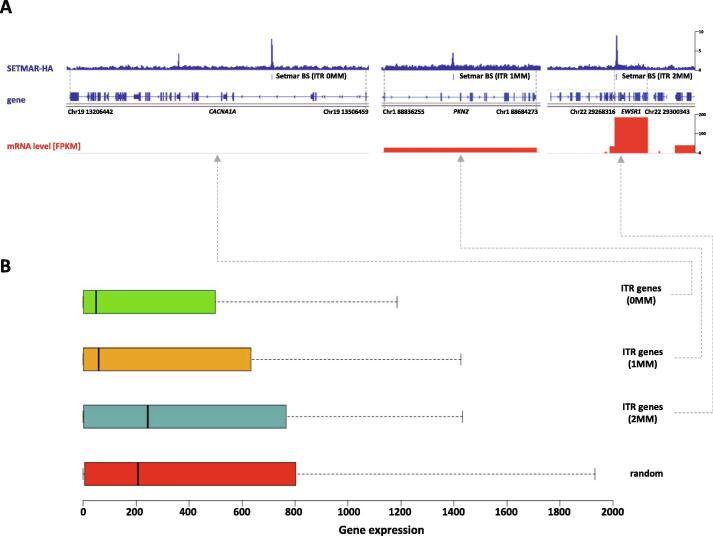
**Gene expression values of SETMAR associated genes scale with the number of ITR mismatches.** (A) Representative genes showing the reciprocal association of mRNA expression level and ITR mismatch number. FPKM stands for Fragments Per Kilobase of transcript per Million mapped reads. (B) mRNA expression levels of SETMAR-HA associated genes grouped by the number of mismatches in ITRs (MM0 = 132, MM1 = 145, MM2 = 29, random = 304). The high fidelity group (MM0) shows significantly reduced mRNA expression levels compared to the random gene group (p-value = 7.5 × 10^-4^, Wilcoxon rank sum test).

To map the chromatin binding sites of SETMAR with high spatial resolution, we set up an experimental system in the nearly haploid HAP1 lymphoblastoid leukaemia cell line [33] in which the endogenous SETMAR locus was knocked out by CRISPR/Cas9 technology followed by complementation by a doxycycline-inducible isoform of SETMAR carrying an N-terminal hemagglutinin tag (pTOV-HA-SETMAR-puro, Fig. 2**A**). The haploid chromosome set allows us to maximize NGS resolution and peak calling accuracy, while knockout of the parental allele is expected to prevent competition between endogenous and epitope-tagged SETMAR isoforms during chromatin binding. Western blot analysis shows that the kinetics of SETMAR-HA induction linearly scaled with the dose of dox concentration, while the tagged protein was not expressed in the absence of drug treatment (Fig. 2**B**) or in wild-type HAP1 cells (Supplementary Fig. S3**A**). The amount of SETMAR-HA at a dox concentration of 0.5 μg/ml was about 3–4 times the amount of endogenous SETMAR expressed in wild-type HAP1 cells (detected by an anti-SETMAR antibody; Supplementary Fig. S3**B**), which provided optimal enrichment for ChIP experiments without significant overexpression of the fusion protein. The observed increase in SETMAR expression levels falls well within the physiological range of SETMAR expression detected in various human tissues and cell lines (Supplementary Fig. S1-S2), which show approximately 80-fold differences. Of the 241 million sequenced reads, we identified 764 / 2228 high-confidence SETMAR-HA binding sites at two significance thresholds (Supplementary Table S1) associated with the 23 chromosomes except the mitochondrial genome (mtDNA), which was used as an internal negative control. Representative binding sites were validated by ChIP-qPCR measurements in dox-treated and untreated samples (Fig. 3 and Supplementary Fig. S4), confirming the specificity of our peak detection. We next analysed the overlap of SETMAR-HA binding sites with annotated genomic categories of the hg38 reference genome (Fig. 4). Functional annotation revealed that most SETMAR-HA sites were located in intergenic regions (52% − 398 peaks) and introns (43% − 329 peaks; Fig. 4**A**), however, the observed frequencies did not differ from the expected (theoretical) distribution. Statistically significant enrichment was observed at transcriptional start sites (TSS; p = 0.03), *MADE1* miniature transposons (p < 2.2 × 10–16), and other, *Hsmar1* transposon-derived ITR sequences (p < 2.2 × 10–16; Fig. 4**B**). The number of peaks in TSS/promoter regions represented only 4% of the binding sites (27 peaks), however, SETMAR-HA was bound to 288 protein-coding genes when TSS-exon–intron-TTS regions were considered (we note that there may be multiple peaks within the same gene). GO-term analysis of SETMAR-associated genes showed enrichment of the MAPK signalling pathway (summarized in Table 1), suggesting a possible role for SETMAR in cell cycle control. Indeed, overexpression of SETMAR significantly reduced the proliferation rate of U2OS osteosarcoma cells [17], consistent with this model. Regarding intergenic regions, all the identified peaks (398 sites) were located in ITR sequences (Fig. 5) or *MADE1* elements flanked by ITRs (Fig. 4**B**). Pileup and Venn diagram analysis (Fig. 5**B-C**) highlights the perfect colocalization between peak summits and ITR motifs within genic and intergenic regions. We note, however, that only a subset of ITRs were accessible for SETMAR binding (1227 motifs − 11.3%), which is still significant compared to a randomized distribution (p < 2.2 × 10–16). The unavailability of ITRs at a given time may be related to local chromatin openness, cis- and *trans*-acting factors, cell cycle stage, or other unknown elements of chromatin structure that have yet to be explored. To address the variance of ITR frequencies and SETMAR binding sites related to chromosome size, we plotted the number of ITRs and SETMAR-HA peaks per chromosome as a function of chromosome length (Fig. 5**C**). The results clearly show that the distribution of SETMAR-HA binding sites and ITR motifs was strongly correlated with chromosome length and showed significant covariation (Spearman r = 0.89; p < 0.001). The X chromosome is a notable exception, as SETMAR binding sites did not correlate with ITR numbers and chromosome size. This unexpected behaviour of chromosome X awaits explanation. To identify critical nucleotide positions in the core ITR motif that are required for SETMAR’s efficient chromatin binding, we grouped ITRs based on the number of mismatches in the 19nt 5′-GGTGCAAAAGTAATTGCGG-3′ sequence (0-3MM groups, Supplementary Table S1) and plotted the SETMAR-HA signal over the categories (Fig. 6). We found that ChIP-seq scores, related to the affinity of SETMAR-HA binding, were inversely proportional to the number of mismatches in the ITR motif (Fig. 6**A**), i.e. the greater the number of mismatches, the lower the affinity of SETMAR (p < 2.2 × 10^-16^). Furthermore, nucleotide positions G2, G4, T14, C17, G18, G19 appeared to be essential for the association of SETMAR and ITRs, as single-nucleotide changes in these bases significantly reduced the affinity of SETMAR-HA binding (Fig. 6**B**). Based on the degree of affinity loss and the prevalence of mutational change, C-to-T and C-to-A transversions at position C17 proved to be the most critical mutations (change in affinity: greater than4-fold; cumulative allele frequency: 33%). Compared to position C17, G-to-A and G-to-T mutations of G18 were also widespread (38%) but did not cause similar affinity changes, while G-to-A mutation of G2 and G4 led to a large decrease in affinity but were rare (2%). Based on the number of mismatches, functional annotation of the ITR categories showed no difference in their genomic localization (Fig. 6**C**). It is noteworthy that more than 60% of the identified mutations were G-to-A and C-to-T changes that correspond to “clock-like” mutation signatures in the COSMIC database [34]. Clock-like mutations are known to form continuously in normal (and cancerous) human cell types, generating mutations at a steady rate throughout the lifetime of cells [35]. Since many ITRs occur in pairs along the chromosomes and one motif is typically of high fidelity (0MM group), the neutral allele is free to mutate during evolution while the conserved motif can still bind and position SETMAR. We found 454 paired ITRs of which 388 (85.4%) were 0MM/1-3MM ITR pairs. In this way, clock-like ITR polymorphisms provide a rationale for fine-tuning SETMAR’s biological function related to transcription. Accordingly, when SETMAR-associated genes were grouped by the number of mismatches in the ITR motif, the high-fidelity group (MM0) showed significantly reduced mRNA expression levels compared to the random gene group (Fig. 7). ITR sequence fidelity was inversely proportional to gene expression levels, i.e., the lower the number of ITR mismatches, the stronger the repression of SETMAR-bound gene loci. The preferential association of SETMAR and repressed genes is fully consistent with previous results [10], which provide strong evidence for SETMAR binding to the most lowly expressed genes with FPKM values between zero and one.

**Table 1:** **Functional annotation of SETMAR-associated genes (n = 288).** Upper table: top five hits of GO-term analysis. Lower table: list of significant MAPK genes.

**#term ID**	**term description**	**observed gene count**	**background gene count**	**FDR**
hsa04010	MAPK signaling pathway	14	293	0.0143
IPR000742	EGF-like domain	15	225	0.0016
GO:0005515	Protein binding	128	6607	0.0409
KW-0597	Phosphoprotein	161	8067	1.90E-05
KW-0025	Alternative splicing	203	10,225	1.13E-08

**Gene name**	**Ensemble ID**	**Function**
ANGPT1	ENSG00000154188	angiopoietin 1
CACNA1A	ENSG00000141837	calcium voltage-gated channel subunit alpha1 A
CACNA2D1	ENSG00000153956	calcium voltage-gated channel auxiliary subunit alpha2delta 1
EFNA5	ENSG00000184349	ephrin A5
FGF1	ENSG00000113578	fibroblast growth factor 1
NTRK2	ENSG00000148053	neurotrophic receptor tyrosine kinase 2
PAK2	ENSG00000180370	p21 (RAC1) activated kinase 2
PLA2G4C	ENSG00000105499	phospholipase A2 group IVC
PRKACB	ENSG00000142875	protein kinase cAMP-activated catalytic subunit beta
PRKCB	ENSG00000166501	protein kinase C beta
SOS1	ENSG00000115904	SOS Ras/Rac guanine nucleotide exchange factor 1
STK3	ENSG00000104375	serine/threonine kinase 3
TGFA	ENSG00000163235	transforming growth factor alpha
MAP3K20	ENSG00000091436	mitogen-activated protein kinase kinase kinase 20

## 4 Conclusions

The results presented in this study clearly show that SETMAR preferentially targets *Hsmar1* transposon ends (ITRs) in living cells that are dispersed throughout the human genome. In contrast to previous studies, we could not detect any off-target binding events at non-ITR sequences. Possible reasons for the differences may include the use of different cell lines (U2OS osteosarcoma cells *vs.* HAP1 lymphoblastic leukaemia cells), tags (FLAG *vs.* HA), NGS platforms (SOLiD *vs.* Illumina), and the low NGS coverage of the previous study [17]. In our experiment, SETMAR was bound to the theoretically expected sequences [10] targeted by its transposase domain. The probability that SETMAR binds to ITR sequences by chance is extremely low (p-value < 2.2 × 10^-16^; Fig. 4**B**). In addition, several ChIP peaks were validated by qPCR in samples with and without doxycycline induction (Fig. 3 and Supplementary Fig. S4), confirming the specificity of ChIP peak detection.

In conclusion, sequence fidelity of the ITR motif has been identified as the only factor that determines the affinity of SETMAR to chromosomes, such that higher ITR fidelity and increased SETMAR chromatin binding resulted in stronger suppression of SETMAR-bound gene loci. This mechanism may be part of a subtle evolutionary strategy to fine-tune transcriptional processes regulated by SETMAR.

## 5 Key points

1. SETMAR/Metnase preferentially targets *Hsmar1* transposon ends (ITRs) in living cells
2. Sequence fidelity of the ITR motif determines the affinity of SETMAR/Metnase to chromosomes
3. Higher ITR fidelity results in increased affinity for chromatin and stronger repression of SETMAR-bound gene loci

## CRediT authorship contribution statement

**Márton Miskei:** Formal analysis, Methodology, Visualization, Data curation. **Adrienn Horváth:** Investigation. **Lívia Viola:** Investigation. **Laura Varga:** Investigation. **Éva Nagy:** Investigation, Validation. **Orsolya Feró:** Formal analysis, Visualization. **Zsolt Karányi:** Software. **Jason Roszik:** Formal analysis. **Csaba Miskey:** Conceptualization, Data Curation, Methodology. **Zoltán Ivics:** Conceptualization, Supervision, Funding acquisition. **Lóránt Székvölgyi:** Conceptualization, Methodology, Supervision, Funding acquisition.

## Declaration of Competing Interest

The authors declare that they have no known competing financial interests or personal relationships that could have appeared to influence the work reported in this paper.
