# Supplementary material for: Genome-wide mapping of binding sites of the transposase-derived SETMAR protein in the human genome
Source: Comput Struct Biotechnol J. 2021 Jul 14;19:4032–41. doi: 10.1016/j.csbj.2021.07.010 (PMC8327481; doi:10.1016/j.csbj.2021.07.010)
Supplement: Supplementary data 1 [file mmc1.pdf]

Supplementary Figures

Genome-wide mapping of binding sites of the transposase-derived SETMAR protein in the human genome

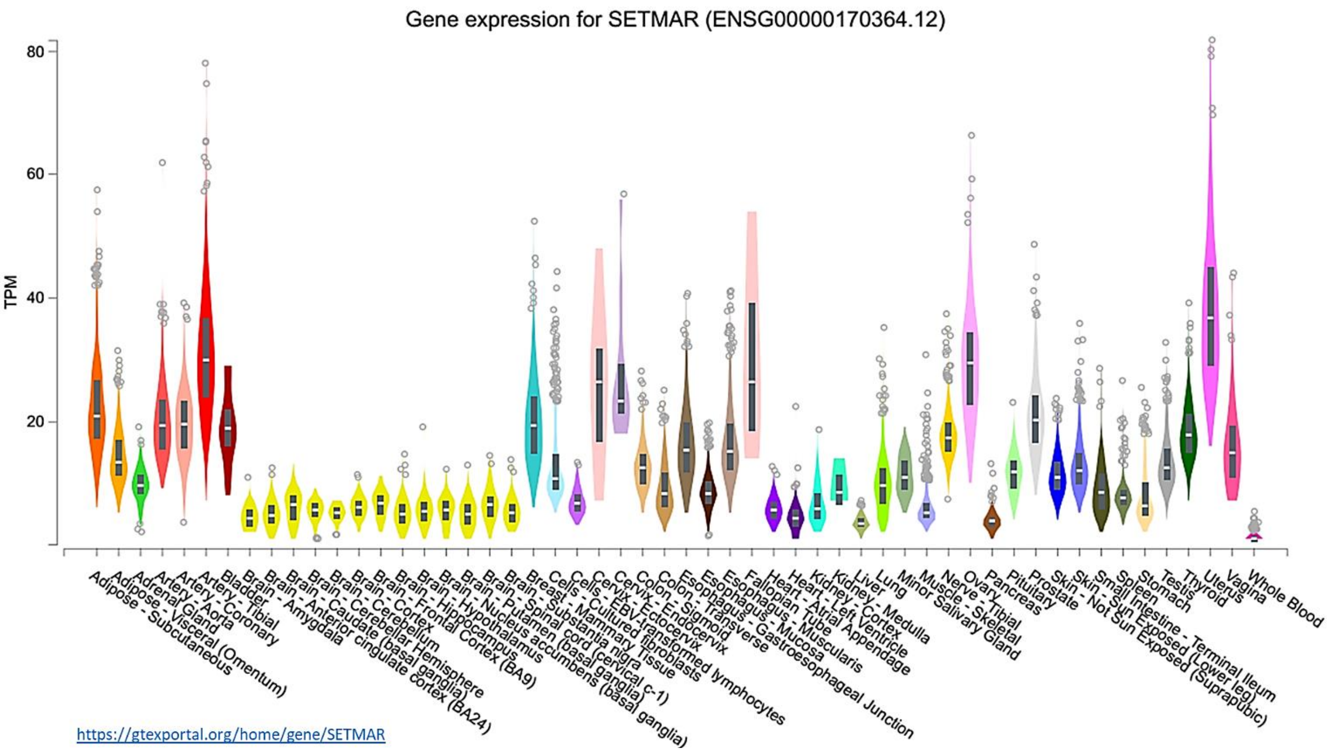

Figure S1. Overview of SETMAR mRNA expression levels in different human tissues. Data are from the GTEx portal. TPM: Transcripts per Million. Boxplots are shown as median and 25th and 75th percentiles. Points are displayed as outliers if they are above or below 1.5x the interquartile range. https://gtexportal.org/home/gene/SETMAR

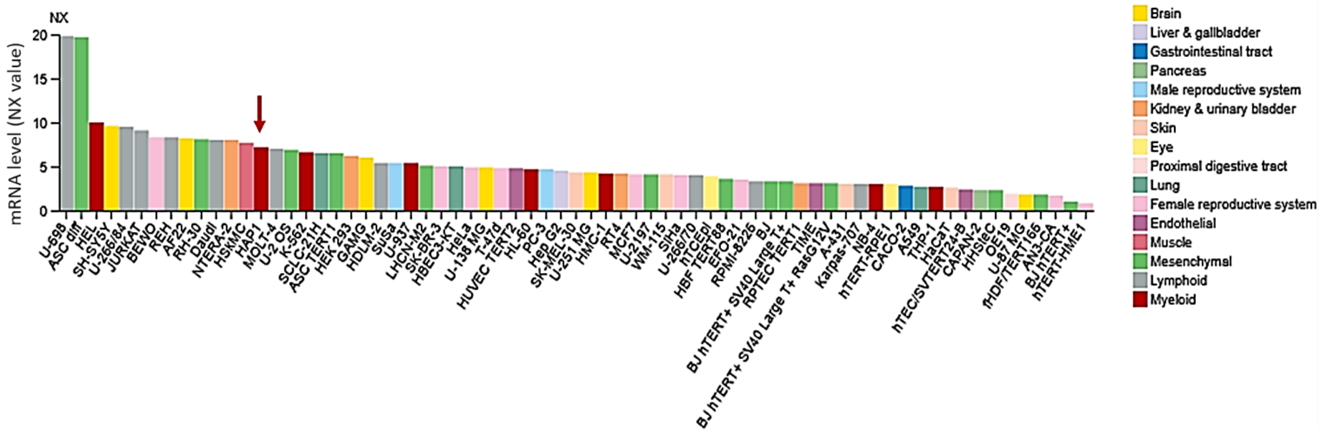

Figure S2. Overview of SETMAR mRNA expression levels in different cell lines analyzed in the Human Protein Atlas. RNA-seq results are reported as normalized NX values. The cell lines are divided into color-coded groups according to their origin in the human body. Vertical red arrow points to the nearly-haploid lymphoblastic leukemia cell line HAP1 used in the current study. https://www.proteinatlas.org/ENSG00000170364-SETMAR/cell

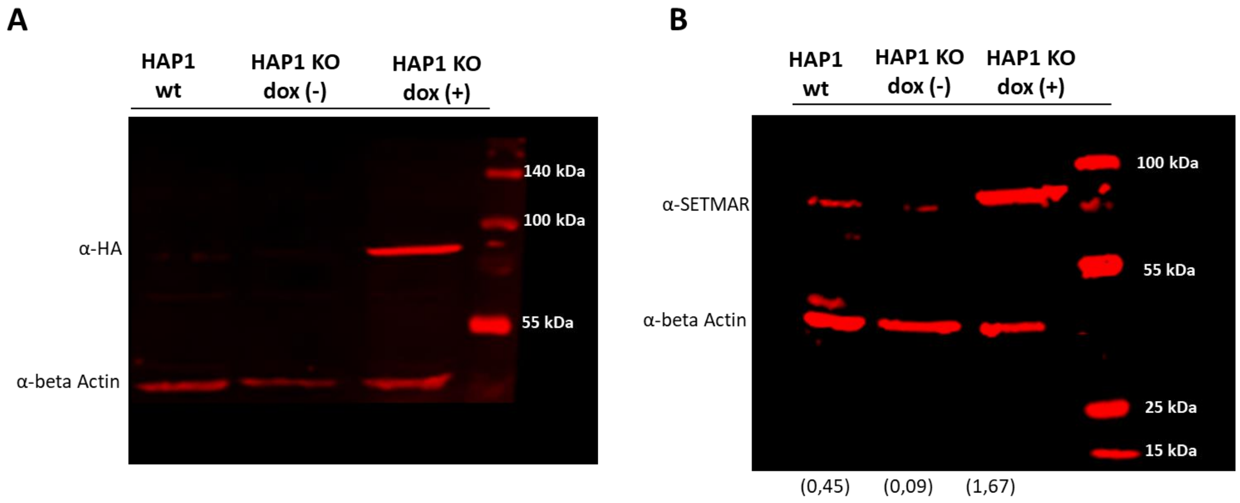

Figure S3. Western blot analysis of SETMAR-HA expression levels (A) and endogenous SETMAR levels (B). Hemagglutinin (HA) and SETMAR signals were detected by anti-HA and anti-SETMAR antibodies, respectively. Dox induction (dox+) was performed at a concentration of 0.5 µg / ml of doxycycline for 24 hours. Loading control: beta Actin. Anti-SETMAR band intensities normalized to loading control (α-SETMAR / α-beta Actin) are shown below panel B.

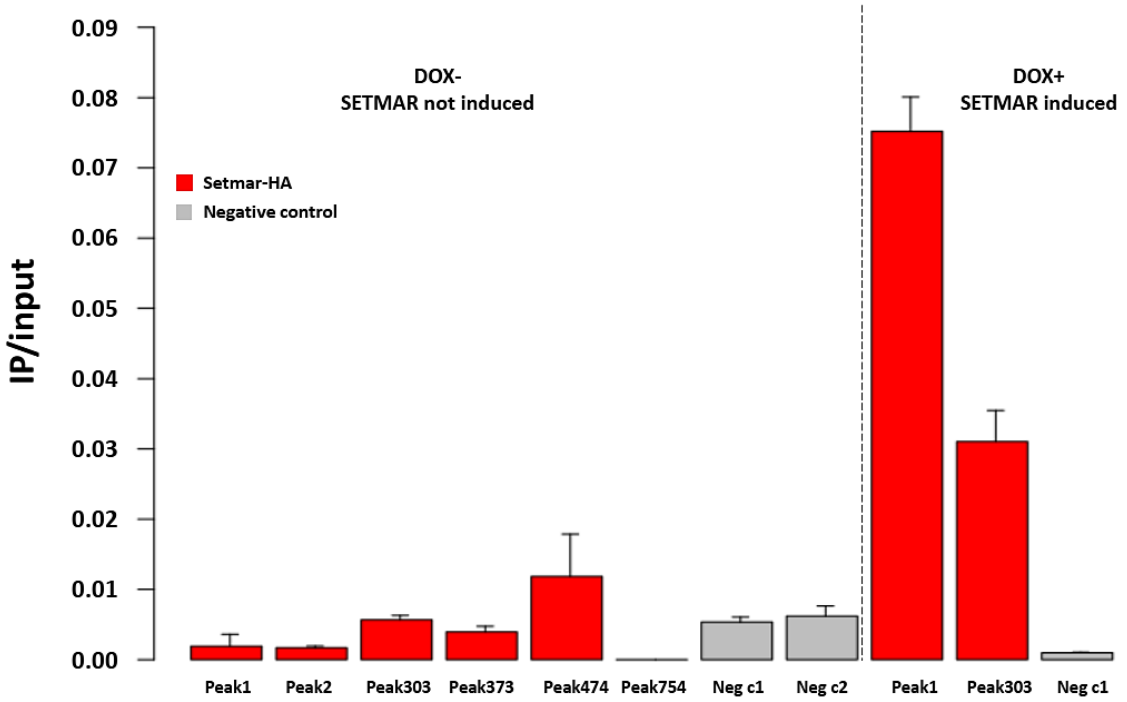

Figure S4. ChIP-qPCR analysis of representative SETMAR-HA binding sites identified by ChIP-seq. In the absence of dox treatment (dox-), SETMAR-HA is not expressed and only background qPCR signal is detected at specific peak positions (red bars). In contrast, high ChIP enrichment is detected in the dox-induced sample (dox+) expressing the SETMAR-HA fusion protein. Grey bars represent negative control sites characterized by a background ChIP-seq signal. Error bars represent the coefficient of variation. Chromosomal position of the test loci is shown in Supplementary Table S1.
